# Transformed Canine and Murine Mesenchymal Stem Cells as a Model for Sarcoma with Complex Genomics

**DOI:** 10.3390/cancers13051126

**Published:** 2021-03-05

**Authors:** Natasja Franceschini, Bas Verbruggen, Marianna A. Tryfonidou, Alwine B. Kruisselbrink, Hans Baelde, Karin E. de Visser, Karoly Szuhai, Anne-Marie Cleton-Jansen, Judith V. M. G. Bovée

**Affiliations:** 1 Department of Pathology, Leiden University Medical Center, 2333 ZA Leiden, The Netherlands; n.franceschini@lumc.nl (N.F.); basverbruggen86@gmail.com (B.V.); A.B.Kruisselbrink@lumc.nl (A.B.K.); J.J.Baelde@lumc.nl (H.B.); A.M.Cleton-Jansen@lumc.nl (A.-M.C.-J.); 2 Department of Clinical Sciences, Faculty of Veterinary Medicine, Utrecht University, 3584 CL Utrecht, The Netherlands; M.A.Tryfonidou@uu.nl; 3 Division of Tumour Biology & Immunology, The Netherlands Cancer Institute, 1066 CX Amsterdam, The Netherlands; k.d.visser@nki.nl; 4 Oncode Institute, Office Jaarbeurs Innovation Mile (JIM), Jaarbeursplein 6, 3521 AL Utrecht, The Netherlands; 5 Department of Cell and Chemical Biology, Leiden University Medical Center, 2333 ZA Leiden, The Netherlands; K.Szuhai@lumc.nl

**Keywords:** sarcoma, complex genomics, mesenchymal stem cells, osteosarcoma, undifferentiated sarcoma, undifferentiated pleomorphic sarcoma

## Abstract

**Simple Summary:**

Sarcomas are rare cancers of mesenchymal origin, the majority of which are characterized by many copy number alterations, amplifications, or deletions. Because of these complex genomics, it is notoriously difficult to identify driver events of malignant transformation. In this study, we show that murine and canine mesenchymal stem cells (MSCs) can be used to model spontaneous malignant transformation towards sarcomas with complex genomics. We show that these MSCs have an abnormal karyotype, many structural variants, and point mutations at whole genome sequencing analysis, and form sarcomas after injection into mice. Our cross-species analysis reveals that p53 loss is an early event in sarcomagenesis, and it was shown that MSCs with a knock-out in *Trp53* transform earlier compared to wild-type MSCs. Our study points to the importance of p53 loss in the transformation process towards sarcomas with complex genomics.

**Abstract:**

Sarcomas are rare mesenchymal tumors with a broad histological spectrum, but they can be divided into two groups based on molecular pathology: sarcomas with simple or complex genomics. Tumors with complex genomics can have aneuploidy and copy number gains and losses, which hampers the detection of early, initiating events in tumorigenesis. Often, no benign precursors are known, which is why good models are essential. The mesenchymal stem cell (MSC) is the presumed cell of origin of sarcoma. In this study, MSCs of murine and canine origin are used as a model to identify driver events for sarcomas with complex genomic alterations as they transform spontaneously after long-term culture. All transformed murine but not canine MSCs formed sarcomas after subcutaneous injection in mice. Using whole genome sequencing, spontaneously transformed murine and canine MSCs displayed a complex karyotype with aneuploidy, point mutations, structural variants, inter-chromosomal translocations, and copy number gains and losses. Cross-species analysis revealed that point mutations in *Tp53/Trp53* are common in transformed murine and canine MSCs. Murine MSCs with a cre-recombinase induced deletion of exon 2–10 of *Trp53* transformed earlier compared to wild-type murine MSCs, confirming the contribution of loss of p53 to spontaneous transformation. Our comparative approach using transformed murine and canine MSCs points to a crucial role for p53 loss in the formation of sarcomas with complex genomics.

## 1. Introduction

Sarcomas represent a large group of mesenchymal tumors with a diverse histological spectrum. Based on histology, ~70 subtypes are recognized [1]. However, based on their molecular alterations, sarcomas can be roughly divided into two groups, where tumors either have simple or complex genomics [2]. Sarcomas with simple genomics are mutation or translocation driven, whereas sarcomas with complex genomics often have multiple alterations such as mutations, translocations, and copy number alterations. 

For many sarcoma subtypes, in particular sarcomas with simple genomics, the molecular pathology has been unraveled in more detail over the last years, which has led to the identification of diagnostic as well as prognostic and predictive biomarkers [2]. In contrast, in tumors with complex genomics, such as osteosarcoma or undifferentiated (pleomorphic) sarcoma, the identification of relevant markers is more difficult. They often have few recurrent alterations, and as a consequence no specific molecular diagnostic markers or targets for therapy are currently available [2,3,4].

Osteosarcoma is a high-grade bone-forming neoplasm that often shows chromoanagenesis (chromothripsis and chromoplexy), in which a single catastrophic event results in fragmentation of chromosomes that are randomly rearranged [1,5,6,7]. Therefore, recurrent alterations in sporadic osteosarcoma are not frequently identified. However, in recent years many next-generation sequencing (NGS) studies have been published showing that alterations in genes such as *TP53* and *RB1* are most common, followed by alterations in *MYC, CCNE1, DLG2, COPS3, PTEN, ATRX,* and *MDM2* [3,5,8,9,10,11,12,13,14,15]. 

Undifferentiated (pleomorphic) sarcoma is a heterogenous high-grade sarcoma that can occur in soft tissue as well as bone, lacking any line of differentiation [1]. It is a diagnosis of exclusion [1]. In undifferentiated (pleomorphic) sarcoma in soft tissue, recent NGS studies identified recurrently altered genes such as *ATRX, RB1, ATM, KDR* and *PIK3CA*, but most often *TP53* [16,17,18,19].

We and others have shown previously that murine MSCs transform spontaneously after long-term culturing, and form sarcoma when injected in mice [20,21,22,23]. These transformed murine MSCs showed many similarities with sarcomas with complex genomics, such as extensive aneuploidy. Unlike murine MSCs, human bone marrow derived MSCs do not transform spontaneously in vitro after long-term culture [24].

In the current study, an alternative approach was used to investigate the initiation of sarcomas with complex genomics by employing not only murine but also canine MSCs. Canines too develop osteosarcoma, with a similar clinical and biological presentation as human osteosarcoma [25,26] and we show here for the first time that canine MSCs can undergo spontaneous transformation.

Using this cross-species approach, the aim was to identify driver genes among the plethora of genetic alterations in sarcoma with complex genomics. Whole genome sequencing was applied to identify single nucleotide variants, structural variants, and copy number alterations. Cross-species analysis revealed that *TP53/Trp53* point mutations are common in late passage canine and murine MSCs, indicating a crucial role for loss of p53 in the initiation of sarcomas with complex genomics.

## 2. Materials and Methods

### 2.1. Mesenchymal Stem Cell Isolation and Cell Culture

Murine bone-marrow-derived mesenchymal stem cells (MSCs) were isolated by collecting the femurs and tibia from surplus C57BL/6J (*n* = 6), NMRI (*n* = 3), FVB mice (*n* = 1) (kindly gifted by Dr. Paul Krimpenfort), or Kcre/P53f (FVB) (*n* = 4) [27] mice between 24–27 weeks old (B6_4, B6_7, B6_10, NMRI_2, NMRI_3, NMRI_9, FVB WT, P53_1, P53_2). Bones were flushed with MSC-medium (αMEM (BE12-169F, Lonza, Switzerland) supplemented with 15% Performance plus Fetal Bovine Serum (16000044, Gibco, Invitrogen Life-Technologies, Scotland, UK), 1% Penicllin-Streptromycin (15140122, Gibco), and 1% Glutamax (35050061, Gibco) and bone marrow cells were resuspended in 75 µL of DNAse (1.33 mg/mL, 11284932001, Sigma-Aldrich, Saint Louis, MO, USA). Cells were washed in 10 mL erythrocyte lysis buffer (NH_4_Cl (8.4 g/L), KHCO_3_ (1 g/L); pH = 7.4, in-house hospital pharmacy). All bone marrow cells per mouse were seeded in one T75 flask, and the medium was refreshed twice per week to wash away non-adherent cells. After 10 days, cells were trypsinized to collect all adherent cells. 

Early passage (P0 or P1) bone-marrow derived MSCs from dogs (canine MSCs) were isolated as described previously [28]. These cells were isolated from the proximal humerus of a Rottweiler (*n* = 1) (OSBMSC1), diagnosed with metastatic conventional osteosarcoma in the distal radius and treated by amputation and chemotherapy, or Labrador Retrievers (*n* = 5) (MSC_492, MSC_490, MSC_447, MSC_446, MSC_405), that were euthanized for unrelated experiments between the age of 2–4 months [29], which was approved by the Ethics Committee of the Utrecht University (DEC 2009.III.06.050). Canine MSCs from Labrador Retrievers were selected based on having positive tri-lineage differentiation capacity, as published elsewhere [30]. Canine MSCs were also cultured in MSC-medium.

All MSCs were cultured at 37 °C with 5% CO_2_ in a humidified incubator, and were tested regularly for mycoplasm. Once cells reached near-confluence (80–90%), cells were passaged with a ratio of 1:3. Every passage, cells were counted with a Bürker-Türk counting chamber to determine cumulative population doublings and any residual cells were frozen.

### 2.2. Transformation Analysis of Mesenchymal Stem Cells

To verify transformation of late passage MSCs karyotyping, soft agar anchorage independent growth assay, Multicolour Combined binary ratio labelling (COBRA) FISH and DNA content analysis were performed.

For karyotyping, late passage canine or murine MSCs were seeded at 6000 cells/cm^2^ in 6-well plates. After cells reached 70–80% confluence, cells were washed twice with serum-free MSC-medium and incubated for 25 min at 37 °C with 1 mL Calyculin A (80 nM, C-3987, LC Laboratories, Woburn, MA, USA). All cells were incubated with 7.5 mL KCl (0.075 M, P9541, Sigma-Aldrich) for 12 min at 37 °C. Cells were then centrifuged 3× for 8 min at 120 rcf. After each centrifugation, the pellet was fixed by adding 8 mL of methanol: acetic acid in a ratio of 4:1. Finally, 12 µL of cell suspension was added on a glass slide inside a humidified chamber (55% humidity at 25 °C). Slides were air-dried and stained with DAPI for microscopic counting of metaphase chromosomes. 

For COBRA FISH, metaphase chromosomes harvested from bone-marrow-derived MSCs (passage 9, during crisis, and passage 10, after transformation) from one C57BL/6J mouse were hybridized using a mouse whole chromosome painting probe set as described in detail previously [31]. Images were collected and analyzed using ColorProc software tool, as described previously [31].

For soft agar anchorage independent growth assay, non-tissue culture treated 6-well plates (351146, Corning, New York, NY, USA) were coated with a bottom layer of 0.7% agarose (dissolved in medium) (16520050, Invitrogen). Late passage canine or murine MSCs were seeded at 20,000 cells per well in 0.35% agarose (dissolved in medium) on top of the bottom layer. Plates were incubated at 37 °C for 3–4 weeks and imaged with GelCount (Oxford Optronix, Milton, United Kingdom).

For DNA content analysis, late passage (P34) and early passage (P1) canine MSCs of the transformed canine MSC culture (OSBMSC1) were analyzed using a standard LSRII (BD Biosciences, San Jose, CA, USA) flow cytometer according to the Vindelov method, without the use of chicken or trout reference nuclei, using the blue 488 nm laser for excitation and a 610/20 nm bandpass filter for collecting propidium iodide (PI) fluorescence. At least 10,000 single cell events were collected using the DNA PI area versus width signals [32].

### 2.3. Whole Genome Sequencing and Data Analysis of Early Passage and Transformed Late Passage MSCs

For whole genome sequencing (WGS), early (OSBMSC1, passage 5 and 6) and transformed late (OSBMSC1, passage 34 and 42) passage canine MSCs, and normal tissue from the Rottweiler were collected for DNA isolation. For murine MSCs, early (B6_4 passage 2, B6_7 passage 2, B6_10 passage 3) and late (B6_4 passage 10, B6_7 passage 15, B6_10 passage 13) passage MSCs from three different C57BL/6J mice were collected for DNA isolation. For DNA isolation, the Wizard Genomic DNA purification kit (A1125, Promega, Madison, WI, USA) was used according to the manufacturer’s instructions. DNA samples were checked for degradation by gel electrophoresis. Whole genome sequencing was performed by BGI Europe using the BGISEQ-500 platform, with 150 bp paired-end reads and 30× coverage. 

Adapters were trimmed from the raw sequencing reads, using cutadapt v1.10 [33] followed by quality trimming with sickle 1.33 [34] with default parameters. The cleaned reads were aligned to the mouse reference genome, GRCm38 (GCA_000001305.2), or to the dog reference genome, CanFam 3.1 (GCA_000002285.2), with bwa mem 0.7.15-r1140 [35] with default parameters. Duplicates were marked with Picard tools 2.9.0 and base quality score recalibration and indel realignment were performed with GenomeAnalysisTK 3.7.0q following GATK Best Practices recommendations [36,37,38].

Somatic SNVs and small Indels for the murine MSCs were identified by comparing the early passage samples for each individual with their respective late passage samples. For the canine MSCs, early passage samples and late passage samples were matched with the normal tissue sample. Three variant callers were applied: Mutect2 v4.0.11.0 [36], Strelka v2.9.10-0 [39] and Varscan v2.4.3 [40], all with default settings, removing those that did not pass the quality filters of the respective callers. The variants were annotated using ANNOVAR (version: 2018-04-16) [41] and filtered for non-synonymous exonic variants with an allele frequency ≥0.2. Human orthologs for *M. musculus* or *C.I. familiaris* genes with variants retrieved from Ensembl Genes 97 using biomaRt v2.38.0 [42]. The human orthologs for each identified variant were called using COBALT [43,44]. Pathogenicity of a variant was determined by ClinVar [45]. For TP53 analysis, transcript variant 1 was used (NM_000546.5).

Copy number variations were identified through Varscan v.2.4.3 copynumber with a minimal segment size of 1000 and maximum segment size of 10,000. Segments were adjusted for GC content by Varscan v.2.4.3 copycaller. Segments were assembled into larger regions of equal log2 ratio using circular binary segmentation with the DNAcopy 1.56.0 [46] package in R 3.5.2 [47]. For canine MSCs, copy ratios were determined by comparing late passage or early passage canine MSCs to normal tissue. For murine MSCs, late passage MSCs were compared to early passage MSCs. Genes spanning multiple segments were assigned the average log2 ratio of the segments. For structural variant calling, we used three different structural variant discovery tools: Delly v0.8.1 [48], manta v2.9.10-0 [49] and lumpy-sv v0.3.0-2 [50]. Structural variants called in the three callers were merged with SURVIVOR v1.0.6 [51], keeping those that were shared by at least two SV callers with a breakpoint window of 1000 bp. The SVs were annotated with SURVIVOR_ant 0.1.0.

Sanger sequencing was used to evaluate the presence or absence of *TP53/Trp53* mutations in DNA samples from all transformed murine MSCs, OSBMSC1 MSCs passage 34, passage 42, and tumor tissue from the same dog. Forward and reverse primers were designed to flank the identified mutation in *TP53* or exons 4, 5, 6, 7 and 8 (Table S4). 

### 2.4. Trilineage Differentiation

Early and late canine or murine MSCs were seeded at 5000 cells/cm^2^ or 15,000 cells/cm^2^ for osteogenic or adipogenic differentiation, respectively. Cells were refreshed with medium twice per week for three weeks. Medium was supplemented with osteogenic differentiation compounds: β-glycerophosphate (5 mM, G6251, Sigma-Aldrich), dexamethasone (0.1 µM, D8893, Sigma-Aldrich), and ascorbate-2-phosphate (0.15 mM, A8960, Sigma-Aldrich), or with adipogenic differentiation compounds: dexamethasone (0.25 µM), ascorbate-2-phosphate (0.15 mM), indomethacin (50 µM, I7378, Sigma-Aldrich), and 1-methyl-3-isobtylxantine (0.5 mM, I5879, Sigma-Aldrich). After three weeks, cells were harvested for RNA isolation or fixed with cold ethanol for 1 h. Alizarin Red S staining solution (2 g Alizarin Red S (02100375, MP Biomedicals, Thermo Fisher Scientific, MA, USA) in 60 mL water, pH 4.2) or Oil Red O staining solution (0.3 g Oil Red (105230, Merck Millipore, Burlington, MA, USA) in 60 mL isopropanol) for osteogenic or adipogenic differentiation, respectively, was added for 5 min and washed with water until solution was clear for imaging. 

For chondrogenic differentiation, late passage canine or murine MSCs were seeded as pellets in a U-shaped 96-well plate (0.5 × 10^6^ cells per well) and cultured in DMEM high glucose (31966, Gibco), supplemented with proline (40 µg/mL, P5607, Sigma-Aldrich), 1% Penicllin-Streptromycin, ITS+ premix (5 µg/mL insulin, 5 µg/mL transferrin, 5 ng/mL selenious acid, 354351, Corning), ascorbate-2-phosphate (0.15 mM), dexamethasone (0.1 µM), TGFb3 (10 ng/mL, 243B3, R&D Systems, Minneapolis, MN, USA), refreshed twice per week. After 4 weeks, cell pellets were fixed with 4% PFA for 30 min, paraffin embedded, and sections were stained with toluidine blue staining solution (0.5 g Azur B (A4043, Sigma Aldrich) in 50 mL MQ, 0.5 g toluidine blue (115930, Merck Millipore) in 25 mL MQ, 0.5 g sodium tetraborate (1330434, Merck Millipore) in 25 mL).

### 2.5. Reverse Transcriptase Quantitative PCR (RT-qPCR)

RNA isolation was done using Trizol (15596026, Invitrogen) according to the manufacturer’s instructions, followed by cDNA synthesis using iScript cDNA Synthesis Kit (1708890, Bio-rad, Hercules, CA, USA) according to the manufacturer’s instructions. Species specific primers for osteogenic markers (*RUNX2, SPARC, SPP1, BGLAP*) and housekeeping genes (*RPL8, B2MG*) were used (Table S4). RT-qPCR was performed using iQ SYBR Green Supermix (1708880, Bio-rad) and a Bio-rad Thermal Cycler according to the manufacturer’s instructions. Relative gene expression levels to housekeeping genes *RPL8* and *B2MG* were determined with the following formula: 2 ^(Ct value housekeeping genes−Ct value gene of interest)^.

### 2.6. Cre-Mediated KO of Trp53 Exon 2–10 in Murine MSCs

Passage 1 MSCs from Kcre/P53f (FVB) mice were seeded with 500,000 cells per T25 flask. The next day, cells were washed twice with PBS and cre-recombinase (3 or 6 µM) (SCR508, Merck-Milipore) was added and incubated for 1 h at 37 °C. Hereafter, cells were washed twice with PBS and replaced with normal MSC-medium. After 2 weeks, DNA was isolated using the Wizard Genomic DNA purification Kit (A1125, Promega) according to the manufacturer’s instructions. PCR was performed using primers flanking the loxP sites in *Trp53* (Table S4). PCR product size was checked by DNA gel electrophoresis. In DNA samples where *Trp53* exons 2–10 are excised, the expected PCR product size is 612 bp, whereas *Trp53* wild-type DNA samples should yield no PCR product.

### 2.7. Western Blotting

Whole cell lysates were made by scraping cells with Hot SDS buffer (1% SDS, 10 mM EDTA, 10 mM Tris pH 7.4) containing protease inhibitor cocktail (11697498001, Roche, Basel, Switzerland) and phosphatase inhibitor cocktail (04906837001, Roche, Basel Switzerland) and incubating lysates for 5 min at 100 °C. Protein concentration of lysates was measured using a microplate reader (Infinite M Plex, Tecan, Switzerland).

Samples (10 µg protein per lane) were loaded on 10% acrylamide gels, and blotted using the Trans-blot Turbo Transfer System (Bio-rad). Blots were blocked in 5% non-fat dry milk in 0.1% Tween-20/PBS for 1 h at RT. Primary antibodies (GAPDH (5174, Cell Signaling, Leiden, The Netherlands), Histon H3 (4499, Cell Signaling), P53 (2524, Cell Signaling) were incubated overnight at 4 °C. After washing blots with 0.1% Tween/PBS, blots were incubated with secondary antibodies, anti-mouse (7076, Cell Signaling) and anti-rabbit (7074, Cell Signaling), for 1 h at RT. Blots were developed with SuperSignal West Pico PLUS Chemiluminescent Substrate (34579, Thermo Fisher Scientific) using the ChemiDoc Touch Imaging System (Bio-rad). Band intensity was calculated using ImageLab software. Protein expression was determined relative to GAPDH. 

### 2.8. In Vivo Tumour Formation

Athymic mice, 6-weeks old, (BALB/c *nu/nu*) were purchased (*n* = 15) from Jackson (Janvier-labs, France), and housed at the animal facility of the Leiden University Medical Center. BALB/c *nu/nu* mice (*n* = 3 per MSC-line) were injected subcutaneously with 50 µL of 0.5 × 10^6^ cells (transformed B6_4, B6_7, B6_10 or OSBMSC1 MSCs) resuspended in PBS/Cultrex BME (1:1 mix) (3433010R1, R&D Systems, Minneapolis MN, USA), under isoflurane anesthetics. Tumor size was measured bi-weekly by calliper. Tumor volume was determined with the following formula: ½ (length × width^2^) [52]. 

Luciferase transduced transformed canine MSCs (OSBMSC1), were injected intratibially with 5 × 10^5^ cells in 10 µL PBS/Cultrex BME (1:1 mix) under isoflurane anesthetics. Every week, tumor growth was measured by non-invasive bioluminescent imaging (BLI) on the IVIS Spectrum Xenogen (Perkin Elmer, Waltham, MA, USA) and quantified In photons/sec/cm^2^/sr after intraperitoneal injection of D-luciferin (150 mg/kg, Synchem UG&CO, Felsberg, Germany). Before the end of the experiment, under injection of anesthetics, microCT (Skyscan 1076 Micro CT scanner, Bruker, Billerica, MA, USA) was performed. Mice were sacrificed by CO_2_ before tumor size reached 1 cm^3^ or after 12 weeks. Tumors, lung, and liver tissues were excised and a half was processed for embedding in paraffin, the other half was fresh frozen. Sections were made of formalin-fixed paraffin-embedded tissue and stained with H&E.

### 2.9. Statistical Analysis

Survival plots were generated using GraphPad Prism 7 software by performing a Kaplan–Meyer analysis. A *t*-test was used to analyze differences in gene expression in samples treated with normal medium or osteogenic medium. To compare densitometry readings of Western blot samples treated with or without cisplatin (3 or 10 µM), an ANOVA test followed by a Dunnett’s test was performed. Comparisons were considered statistically significant using a significance level of 5%. 

## 3. Results

### 3.1. All Murine MSCs Transform Spontaneously after Long-Term In Vitro Culture

**Figure 1:**
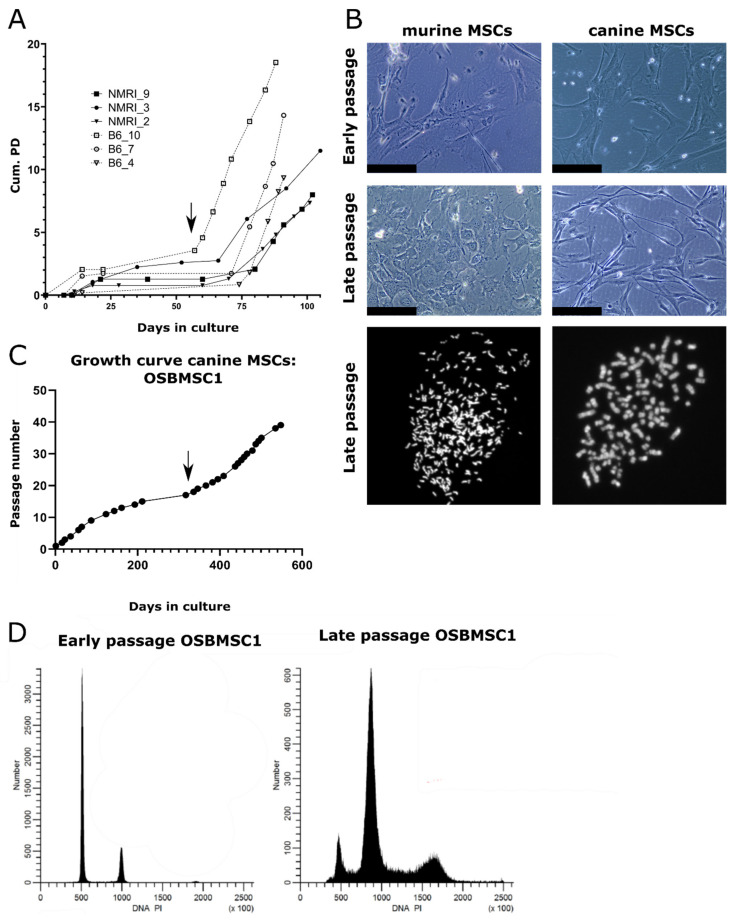
Mesenchymal stem cells (MSCs) from dogs and mice transform spontaneously after long-term culture. (**A**) MSCs isolated from NMRI or C57BL/6J mice undergo spontaneous transformation after long-term in vitro culture based on cumulative population doubling (Cum. PD). Example of transformation event in B6_10 is indicated by an arrow. (**B**) Representative images of murine and canine MSCs. Late passage murine MSCs show a higher nuclear to cytoplasm ratio, as evident from the nuclear enlargement, compared to early passage MSCs. Scalebar represents 100 µm. Karyotyping showed late passage MSCs have an increased number of chromosomes per cell after DAPI staining, as murine and canine cells normally have 40 or 78 chromosomes, respectively. (**C**) MSCs isolated from a 7-year-old Rottweiler transform spontaneously after long-term ex vivo culture (OSBMSC1), indicated with an arrow, at each data point, cells were trypsinized and passaged. (**D**) DNA content analysis by flow cytometry shows that late passage canine MSCs OSBMSC1 have become aneuploid.

Bone-marrow derived MSCs were isolated from two strains (C57BL/6J and NMRI) and differentiation capacity towards the osteogenic and adipogenic lineage was evaluated. There were two out of three MSC cultures that showed both osteogenic and adipogenic differentiation capacity, while the third (B6_4) only showed osteogenic differentiation (Figure S1). Due to the limited amount of early passage untransformed cells, not all differentiation assays could be performed for all cultures. Long-term cultured MSCs underwent spontaneous transformation after 57–74 days, which was accompanied by an increased growth rate (Figure 1A) and morphological changes: late passage murine MSCs have an increased nuclear to cytoplasm ratio compared to early passage murine MSCs (Figure 1B). We could confirm the transformation by karyotyping of all late passage murine MSCs, as most late passage cells harbored typically between 74–216 chromosomes, whereas early passage cells mostly have the normal amount of 40 chromosomes (Figure 1B). Soft agar assay demonstrated anchorage independent growth in three out of six late passage murine MSC cultures (Figure S2).

### 3.2. Infrequent Spontaneous Transformation of Canine MSCs after Long-Term In Vitro Culture

Not only murine MSCs, but also one MSC culture derived from the seven-year-old Rottweiler (OSBMSC1) escaped a crisis phase after long-term in vitro culture, followed by rapid cell growth (Figure 1C). However, the spontaneous transformation of canine MSCs seemed to be a rare event, as we did not observe transformation in the five other canine MSC cultures isolated from the Labrador Retrievers (Figure S3). Similar to the murine MSC late passage cultures, late passage MSCs of OSBMSC1 showed an abnormal chromosome number between 91–100 chromosomes, whereas normal chromosome number should be 78 (Figure 1B). Morphological changes in transformed canine MSCs were less evident compared to transformed murine MSCs (Figure 1B). Aneuploidy in the late passage canine MSCs from OSBMSC1 was also evident from DNA content analysis by DNA flow cytometry (Figure 1D). 

### 3.3. Clonal Expansion of Murine MSCs Prior to Transformation Event

**Figure 2:**
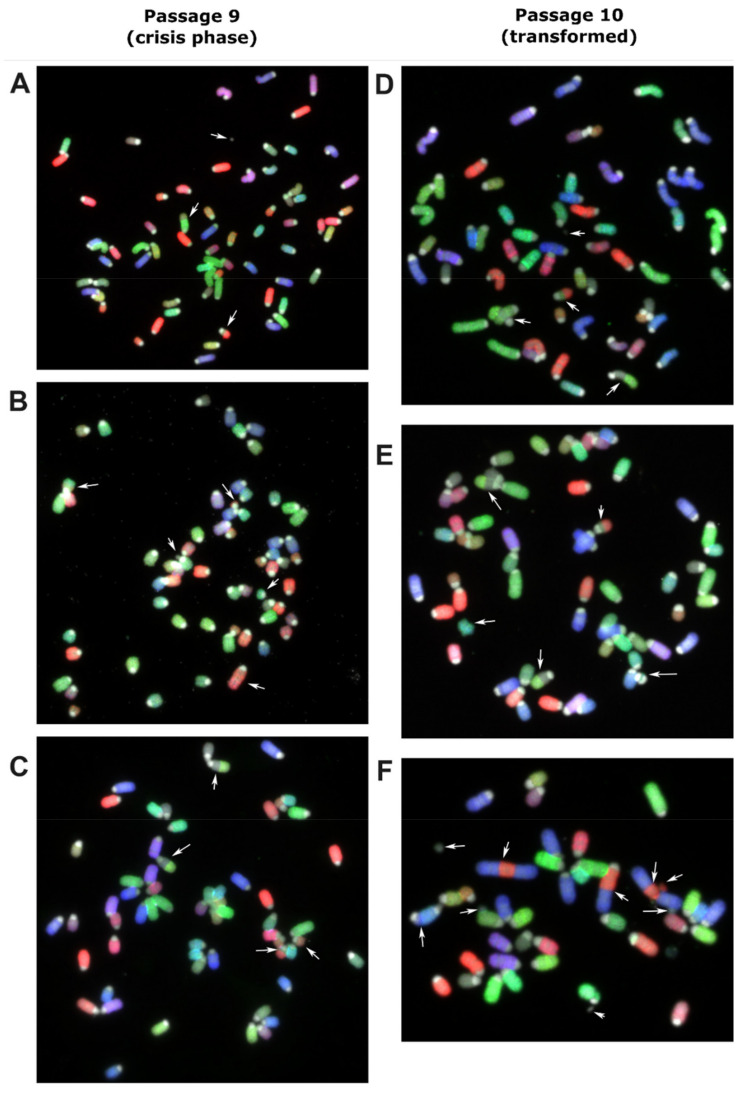
Murine MSC derived from bone marrow of a C57BL/6J mouse was harvested and molecular karyotyped at crisis phase at passage 9 (**A**–**C**) and the next passage (P10) after transformation (**D**–**F**) using COBRA-FISH. In all panels, both numerical and structural chromosomal alterations are visible. Arrows indicate involved chromosomes and rearranged chromosomes are listed per panel from top to bottom and from left to right: (**A**) Centromeric fragment, der(4)t(4;7), der(7)t(3;7), (**B**) der(16)t(13;16), del(3), centromeric fragment, del(13), der(3)t(3;12), (**C**) der(18)t(4;18), der(18)t(4;18), del(3), der(7)t(3;7), (**D**) centromeric fragment, der(7)t(3;7), der(18)t(4;18), der(18)t(4;18), (**E**) der(7)t(3;7), der(18)t(4;18), ace(17), der(18)t(4;18), dic(17;17), (**F**) centromeric fragment, acentric chromosome from chromosome 2-3-2 fusion, der(3)t(2;3), acentric chromosome from chromosome 2-3-2 fusion, fragment of chromosome 3, centromeric fragment, centromeric fragment, dic(5;11), centromeric fragment. COBRA-RGB-color images were superimposed with DAPI (grey) to visualize chromosome centromeric regions. Magnification 630×.

The transformation event likely occurred during a period where no expansion of cells was observed, (lag-phase of Figure 1A). MSCs derived from bone marrow of one C57BL/6J mouse were harvested and molecular karyotyped by COBRA-FISH at the crisis phase at passage 9. There were 19 cells suitable for analysis and showed a ploidy of 3n (Figure 2A–C). Eighteen of the nineteen cells showed structural chromosomal rearrangements, deletions and/or translocations and the presence of centromeric fragments. Random structural changes involving deletion and translocations were observed in all chromosomes except for 17, 19, and X, with alterations in chromosomes 3, 7, 6, and 4 being the most frequent. One cell showed the karyotype: 3n, XXX, der(18)t(4;18)x2, del(3), der(7)t(3;7) (Figure 2C). This karyotype was also seen in the next passage (passage 10) after crisis, in which 15/22 cells showed clonal expansion of a dominantly 3n cell population carrying der(18)t(4;18),der(7)t(3;7) (Figure 2D–F). From these 15 cells, 7 had additional alterations, indicating the formation of subclones. Within the non-clonal cells, one cell was identified with a complex chromosome break, chromosome rearrangement, and acentric fragment, indicating the presence of genomic instability (Figure 2F).

### 3.4. Transformed Murine and Canine MSCs Display Variable Mesenchymal Differentiation Capacity

**Figure 3:**
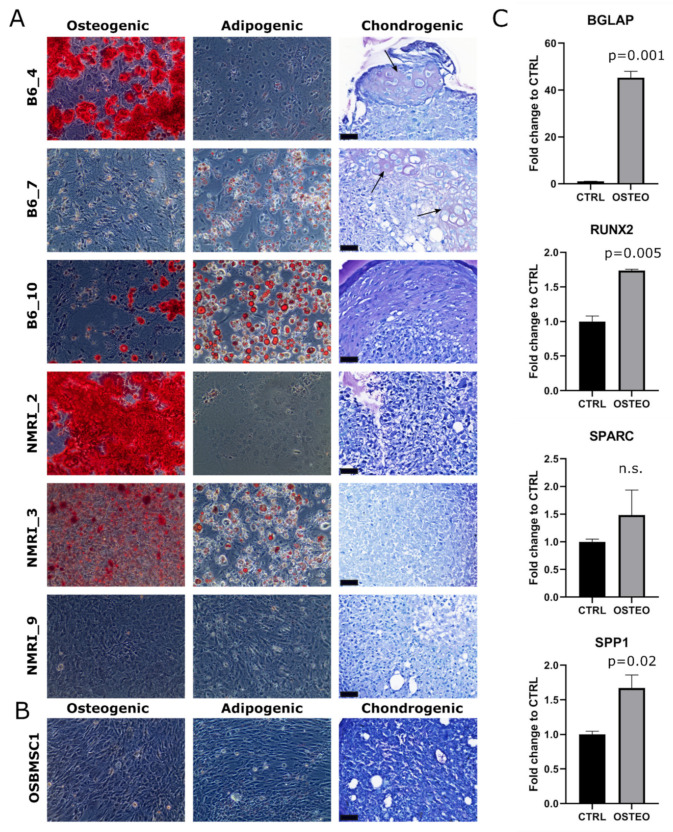
Trilineage differentiation of late passage murine and canine MSCs. Osteogenic, adipogenic and chondrogenic differentiation of (**A**) late passage murine MSCs and (**B**) late passage canine MSCs of OSBMSC1 was assessed by Alizarin Red (Magnification 100×), Oil Red O (Magnification 100×), and Toluidine Blue staining, respectively, and was highly variable among donors. For Toluidine Blue staining, arrows indicate metachromatic staining, indicative for cartilaginous matrix. Scale bar represents 40 µm. (**C**) Osteogenic gene expression markers (*BGLAP, RUNX2, SPP1*) were significantly upregulated in late passage OSBMSC1 treated with osteogenic stimuli for 3 weeks (OSTEO), compared to non-treated MSCs (CTRL). Bars represent the mean of one experiment performed in triplicate ± standard deviation.

The trilineage differentiation capacity of transformed late passage murine MSCs was highly variable: four out of six murine MSC cultures (B6_4, B6_10, NMRI_2, NMRI_3) could differentiate towards the osteogenic lineage, three out of six cultures (B6_7, B6_10, NMRI_3) could differentiate towards the adipogenic lineage, and two out of six cultures (B6_4 and B6_7) showed chondrogenic differentiation (Figure 3A). The transformed canine MSCs did not show any trilineage differentiation capacity under the conditions studied (Figure 3B), but by RT-qPCR analysis, most osteogenic markers (*BGLAP*, *RUNX2*, and *SPP1*) were significantly upregulated (Figure 3C). 

### 3.5. Transformed Murine MSCs Form Tumours with Variable Growth Rate and Histology In Vivo

**Figure 4:**
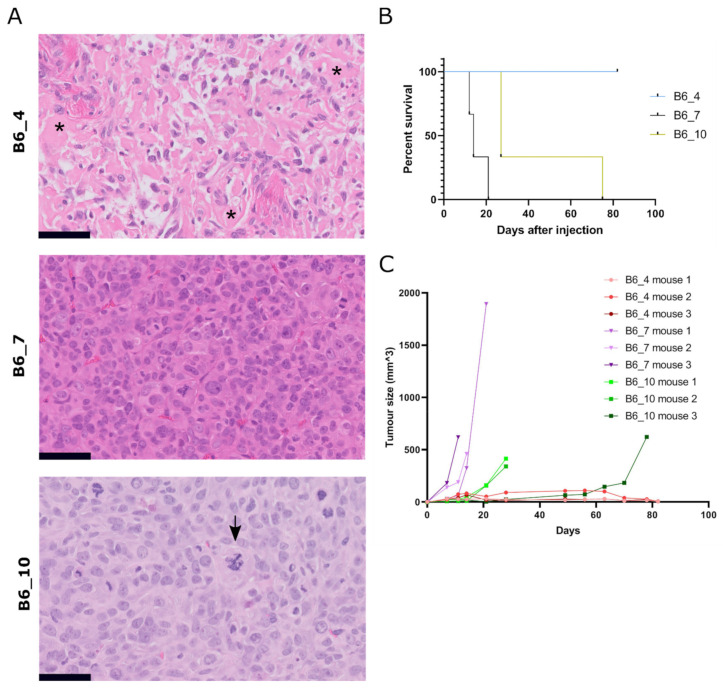
Subcutaneous injection of transformed murine MSCs result in tumor formation with variable histology. (**A**) H&E staining of tumors formed after subcutaneous injection of transformed murine MSCs (B6_4, B6_7, B6_10). Tumor B6_4 displayed a moderately cellular and pleomorphic tumor with deposition of amorphous eosinophilic extracellular matrix, suggestive of osteoid (indicated with asterisks). In contrast, tumors B6_7 and B6_10 were more cellular, pleomorphic, with a high number of mitoses, and morphologically lack any differentiation. Atypical mitosis were present, indicated with an arrow in B6_10. Tumor volumes for B6_4, B6_7 and B6_10 were 0.005 cm^3^ (*n* = 2), 0.4–1.1 cm^3^ (*n* = 3) and 0.3–0.6 cm^3^ (*n* = 3), respectively. Scalebar represents 50 µm. (**B**) Number of days after subcutaneous injection when mice were sacrificed due to an increase in tumor volume. (**C**) Tumor size measured by calliper of each mouse after subcutaneous injection of transformed murine MSCs (t = 0).

To confirm tumorigenicity of transformed MSCs, we injected three (B6_4, B6_7, B6_10) transformed murine MSCs and the transformed canine MSCs (OSBMSC1) subcutaneously in mice, in triplicate. Within 21–82 days, eight out of nine mice that were injected with transformed murine MSCs developed tumors (Figure 4A–C). No metastases were found in liver or lung tissue. All mice injected with MSCs from B6_7 and B6_10 had to be sacrificed due to tumor formation, but mice injected with MSCs from B6_4 were only sacrificed at the end of the study (81 days). However, numbers were too small to conduct meaningful statistical analysis. (Figure 4B). The growth rate, tumor size, and histological spectrum of tumors was variable between the lines. A total of two out of three mice injected with murine MSCs B6_4 developed very slow growing and small tumors (0.005 cm^3^). Both tumors were moderately cellular, pleomorphic, with deposition of an amorphous eosinophilic matrix strongly suggestive of osteoid, thereby resembling human osteosarcoma (Figure 4A). Interestingly, this MSC culture did not show any colonies with the soft agar assay (Figure S2). On the contrary, all mice injected with murine MSCs B6_7 developed the fastest growing and largest tumors (0.4–1.1 cm^3^) after 12–21 days and showed colonies with the soft agar assay. All mice injected with MSCs from B6_10 developed tumors after 27–74 days, and all tumors were similar in size (0.4–0.6 cm^3^) and histology as the tumors from B6_7. Histologically, all six tumors from B6_7 and B6_10 showed a highly cellular, pleomorphic and mitotically active proliferation of large undifferentiated cells, suggesting resemblance to human undifferentiated (pleomorphic) sarcoma. Mice that were injected with transformed canine MSCs, either subcutaneously or intratibially, did not develop any tumors. We did observe luciferase activity after intratibial injection, supporting initial engraftment, but this signal decreased over time.

### 3.6. Transformed Murine and Canine MSCs Have Numerous Structural Variants and Copy Number Alterations

**Figure 5:**
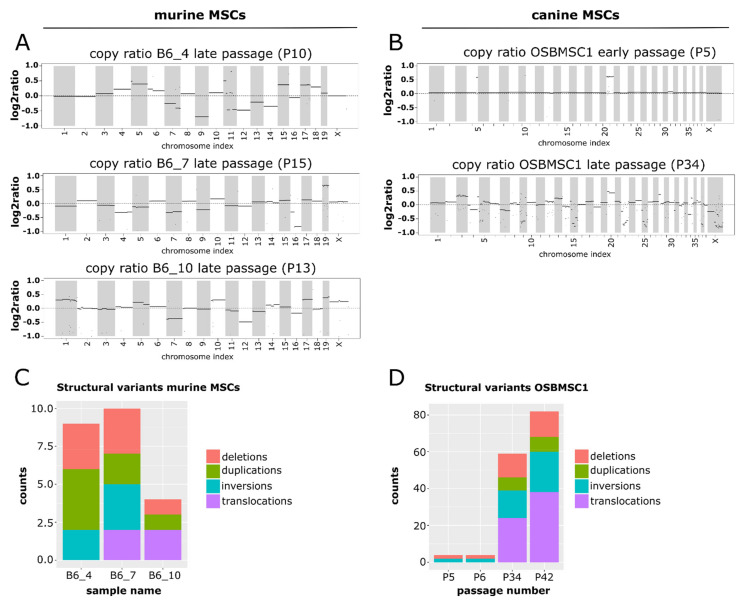
Late passage murine and canine MSCs show a complex molecular karyotype after whole-genome sequencing. Segmented copy ratios are shown for (**A**) late passage murine MSCs and (**B**) early and late passage canine MSCs. Positive copy ratios indicate a gain of genomic materials, whereas negative ratios indicate losses. The total number of structural variants in (**C**) late passage murine MSCs and (**D**) early and late passage canine MSCs.

To study the complex genomics in transformed murine and canine MSCs in more detail, copy number alterations and structural variants were determined for three murine MSC cultures (B6_4, B6_7 and B6_10), by comparing late passage MSCs with early passage MSCs, and the canine MSC culture (OSBMSC1), by comparing canine MSCs with normal tissue from the same dog, using WGS. For murine MSCs, all transformed late passage samples show numerous copy number alterations across the entire genome (Figure 5A). Although late passage MSCs from B6_7 harbored a large deletion in chromosome 4, including the *Cdkn2a* and *Cdkn2b* gene (Table S1), no recurrent copy number alterations were found. Furthermore, no deviations in whole chromosomes were identified in early passage murine MSCs (Figure S4).

For canine MSCs, in the early passage MSCs there were two apparent copy number alterations: a duplication of chromosome 20, and a small regional duplication in chromosome 4 (Figure 5B). After transformation, aneuploidy was apparent in the copy ratio plots. The copy ratios for P34 show that the abnormalities in chromosomes 4 and 20 were retained, but in addition a myriad of other chromosomes showed changes in copy ratio (Figure 5B). 

For murine MSCs, the number of structural variants was limited (Figure 5C). No recurrent structural variants were identified between the different mice. Furthermore, the number of variants varied between the different samples. This was apparent in the number of inter chromosomal translocations in the samples, with no translocations detected in B6_4, only one translocation in B6_7 between chromosome 9 and X, and translocations between chromosome 1 and 5 and chromosome 3 and 7 in B6_10 (Figure S5A). 

For canine MSCs, the overall number of structural variants increased dramatically after MSC transformation (Figure 5D). Whereas the structural variations in the early passages were limited to two deletions and two inversions, in the late passage, samples a progression in the number of structural variants was observed. The increase in structural variations like translocations highlight the emergence of a complex karyotype upon MSC transformation (Figure S5B). However, structural variants found in transformed canine MSCs were not observed in the orthologous genomic regions in transformed murine MSCs. 

### 3.7. Cross-Species Analysis Reveal TP53/Trp53 Mutation as a Common Single-Nucleotide Variant in Transformed Murine and Canine MSCs

To search for common driver alterations (SNVs) that may play a role in the initiation of sarcomas with a complex genome, whole genome sequences from three murine and one canine MSC line, before and after spontaneous transformation, were compared. 

**Figure 6:**
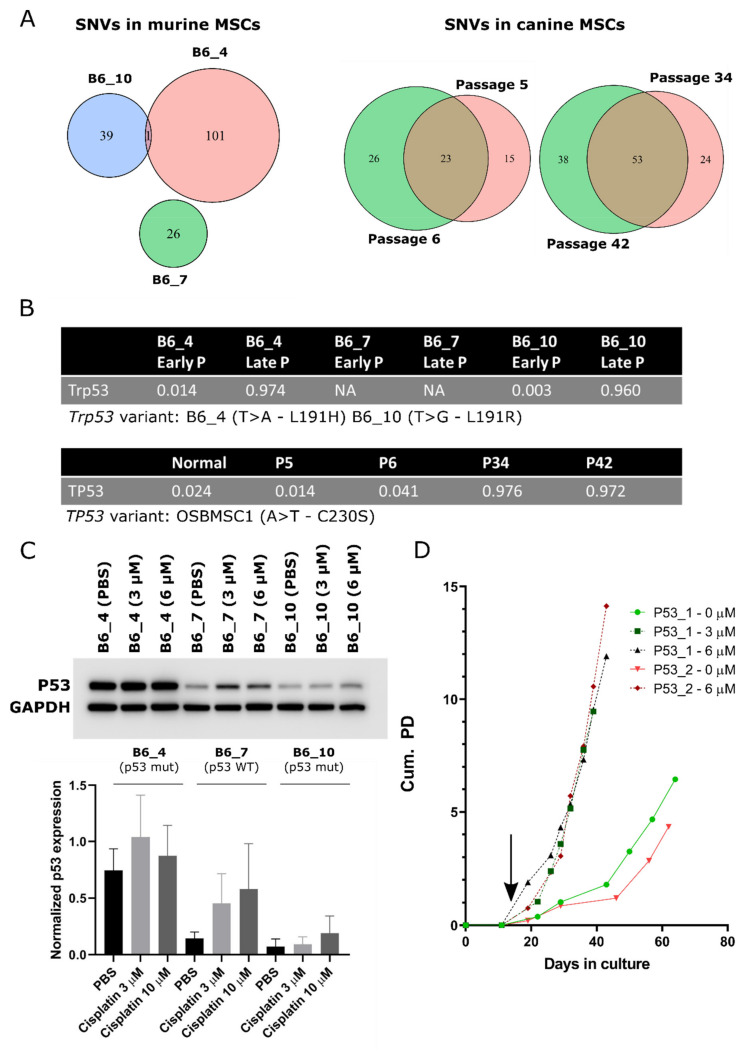
Point mutations in *TP53/Trp53* are a common single nucleotide variant (SNV) in transformed murine and canine MSCs. Venn diagrams depicting the total number of SNVs and small indels in (**A**) late passage murine MSCs and early and late passage canine MSCs. (**B**) Single nucleotide variant in *Trp53* or *TP53* was the SNV with the highest allele frequency in late passage murine and canine MSCs. (**C**) Western blot showing p53 protein expression in late passage murine MSCs, treated with PBS or cisplatin (3 and 10 µM). Murine MSCS with WT p53 (B6_7) increase p53 expression over 3-fold, whereas murine MSCS with mutant p53 (B6_4 and B6_10) increase p53 expression only 1.2–2.1 fold, indicating p53 function is partially impaired in these cells. GAPDH was used as a loading control. Quantification of relative p53 protein expression to GAPDH is depicted on the bottom panel. Statistical analysis was performed based on three independent experiments, but here only one representative blot is shown. No changes in protein expression were statistically significant. Whole blots with densitometry readings can be found in Figure S9. (**D**) Murine MSCs from Kcre/p53f mice were treated with cre-recombinase (3 or 6 µM) at day 11 (indicated by black arrow) inducing immediate transformation, whereas non-treated MSCS transform later. At each data point, cells were trypsinized and counted to calculate cumulative population doubling (Cum. PD).

The SNV with the highest allele frequency in late passage murine MSCs of mouse B6_4 and B6_10, and the only SNV overlapping between different mice (Figure 6A and Table S2), was a non-synonymous point mutation in *Trp53*. This point mutation in murine MSCs was a T > A/L191H and a T > G/L191R variant in mouse B6_4 and B6_10, respectively (Figure 6B). This mutation was confirmed by Sanger sequencing in B6_4 and B6_10 transformed MSCs (Figure S6), which was not present in B6_7. No other mutations in *Trp53* in the same exon or in the most commonly mutated [53] exons, 4, 5, 7, and 8 were identified in other transformed murine MSCs (NMRI_2, NMRI_3, NMRI_9) (Figure S6). Using COBALT [43], the identified variants were shown to be orthologous to the human *TP53* variant L194H or L194R, both reported to be likely pathogenic according to ClinVar. As cisplatin treatment causes DNA damage and activates the p53 pathway, functionality of p53 was assessed by treating murine MSCs with PBS or cisplatin (3 or 10 µM). MSCs from B6_4 and B6_10 treated with cisplatin only slightly increased p53 expression (1.2–2.1 fold). This was in contrast with MSCs from B6_7, where cisplatin treatment increased the expression of p53 over 3-fold (Figure 6C), although this was not statistically significant. 

In late passage canine MSCs, we identified more SNVs and small indels compared to early passage MSCs (Figure 6A). In the transformed MSCs, the variant with the highest allele frequency was in *TP53*, with an allele frequency of almost 1, indicating loss of the WT allele (Figure 6B and Table S3). This was an A668T/C230S variant. C230 is located in the *TP53* DNA-binding domain and as the amino acid introduces a cysteine it is likely that the variant has a negative impact on the functioning of p53. Using COBALT, this variant is orthologous to the human *TP53* variant C242S and reported to be likely pathogenic according to ClinVar (rs1057519982). As the canine MSCs have been isolated from a dog with osteosarcoma, the question remained whether the transformed cells could originate from a micrometastasis from the original tumor. The *TP53* mutation we identified in the transformed/late passage MSCs was absent in the tumor and in the healthy tissue of the dog (Figure S7) and has not been identified previously in NGS studies of osteosarcoma in canine patients [54,55]. 

Aside from *TP53*, the only gene with a variant that was present at an allele frequency over 0.75 in both P34 and P42 OSBMSC1 is *UNC80* C1145G/P382R. The protein encoded by *UNC80* is part of the sodium leak ion channel (*NALCN*) [56]. Variants in *NALCN* and *UNC80* have been linked to many diseases, including several types of cancer [56,57]. However, the P382R variant is not located in any Pfam or InterPro annotated domains, therefore its impact on protein function is more difficult to assess. 

### 3.8. Murine MSCs with a KO of P53 Transform Earlier Compared to WT Murine MSCs

As both murine and canine transformed MSCs had acquired *TP53/Trp53* alterations upon transformation, we further investigated the role of *TP53/Trp53* in spontaneous malignant transformation. We created a KO of *Trp53* in murine MSCs, in which exon 2–10 are flanked by Lox-sites, by in vitro treatment with cre-recombinase. We observed that MSCs treated with 3 or 6 µM cre-recombinase, transformed immediately after treatment, whereas non-treated murine MSCs transformed after 60 days, comparable with MSCs from mice of other WT strains (Figure 1A and Figure 6D). KO of *Trp53* was confirmed by Western blotting and PCR (Figure S8A,B). Transformed *Trp53* KO MSCs showed a complex karyotype with aneuploidy (Figure S8C). To exclude that cre-recombinase treatment itself has an effect on cell expansion, we treated MSCs from FVB WT mice with cre-recombinase, which did not have an effect on expansion rate (Figure S8D). These results confirm that p53 plays an important role in the spontaneous transformation of MSCs and the generation of a complex genome.

## 4. Discussion

The pathogenesis of sarcomas with complex genomics is notoriously difficult to study, especially if no benign precursor is known, e.g., for osteosarcoma. Here we use MSCs from mice and a dog that spontaneously transform in vitro, to pinpoint, among the huge amount of genetic alterations, those genes, including *TP53*/*Trp53*, that are involved in the initiation of sarcomas with complex genomics. 

There is an interesting difference between species, where human MSCs never transform in vitro [24], canine MSCs occasionally transform, and murine MSCs always show a spontaneous transformation. We and others have shown that all bone-marrow-derived murine MSCs, independent of strain or age, eventually transform [20,21,22,23]. For the first time, we now also observed spontaneous transformation in canine MSCs. In contrast to murine MSCs, the phenomenon is rarer in canine MSCs, as only one out of six canine MSC cultures transformed spontaneously, even though cultures were kept for over 150 days. Of note, the canine MSCs that transformed were isolated from an old Rottweiler (7 years) with osteosarcoma, whereas the other donors were Labrador Retrievers, much younger (4–5 months) and without osteosarcoma, so transformation of MSCs in dogs might be age and breed dependent. Furthermore, we excluded that the transformed MSC cultures from the Rottweiler originated from a micrometastasis from the osteosarcoma, as the TP53 mutation that was found in the transformed canine MSCs, was absent in the primary tumor, although we cannot completely rule out that Sanger sequencing missed a subclonal presence of the mutation in the tumor. The difference in transformation between species might also be explained by inbreeding. The mice used in this study were inbred mouse strains, resulting in a more homozygous genome, such that it could possibly cause more frequent spontaneous transformation compared to dogs or humans.

After subcutaneous injection of transformed murine MSCs, mice formed tumors with variable growth rate and histology. The tumors with a slower growth rate showed osteogenic differentiation, and are histologically identical to human high- grade osteosarcoma. The faster growing tumors were highly cellular and pleomorphic, morphologically lacking any line of differentiation, suggesting resemblance to human undifferentiated (pleomorphic) sarcoma, although immunohistochemical markers commonly used to rule out specific lines of differentiation, for instance myogenic differentiation, could not be applied on the mouse tissue because these antibodies are of mouse origin, rendering stainings non-specific. As the histology of the tumors is diverse after subcutaneous injection, it is possible that additional (epi)genetic factors determine the definitive histological subtype or that it is determined by the micro environment, which would have to be studied in an orthotopic model. In contrast to murine MSCs, the transformed canine MSCs did not form tumors in athymic mice, both subcutaneously and intratibially. It might be that canine cells have difficulty engrafting in a mouse microenvironment, however, previously published studies reported the successful growth of (adipose-derived) MSCs and osteosarcoma cell lines from canine origin in immune deficient mice, both subcutaneously and intratibially [58,59]. 

Transformed murine and canine MSCs show translocations, copy number alterations, and an increased number of single nucleotide variants and structural variants in later passages, resembling the genomic alterations in sarcomas with complex genomics. By COBRA-FISH karyotyping, we identified that prior to the transformation event, cells showed both numerical and structural changes with non-clonal random rearrangements and a strikingly high number of centromeric fragments indicating major loss of chromosomal material. After the crisis phase, a clonal expansion of a successor clone was seen with further rearrangements, but with non-clonal variants. In the non-clonal related cells, however, a great genomic instability was depicted by observing chromosomal breaks, chromosomal rearrangement, and acentric fragments that would be leading to further genomic rearrangement or could be lethal for the next cell generation. 

The cross-species approach, described in the present study, enabled us not only to investigate single cells during the crisis phase, but also to compare earlier non-transformed passages of the same mouse or dog with cells after transformation, creating the opportunity to identify genes and pathways involved in the formation of sarcomas with complex genomics. We identified point mutations in *TP53/Trp53*, with loss of heterozygosity in the late passages. The important role of p53 in spontaneous transformation is further evident from the murine MSCs that transformed immediately after the induction of a deletion of exon 2–10 of *Trp53*. Taken together, our unique cross species model has identified an important role for *TP53* in the formation of sarcomas with complex genomics. The importance of *TP53* for the malignant transformation process has been found in other cancer types [60,61,62]. Our results are also in line with the hypothesis [63] that *TP53* mutations are early events and selected for in ectodermal and mesodermal-derived tumors, such as sarcomas, in contrast to tumors from endodermal origin, such as colon cancer, in which *TP53* mutations are the last to occur. 

In sarcomas with complex genomics, such as undifferentiated sarcoma and osteosarcoma, alterations in *TP53* and/or the p53 pathway are frequently found: 22–66% in UPS and 47–90% in sporadic osteosarcoma [5,9,10,17,18,64]. Previous studies have shown a link between *TP53* alterations and chromoanagenesis, characteristic for complex genomics sarcomas such as osteosarcoma, but the precise mechanism is unknown [7,65,66,67]. *TP53* alterations are not the only causative factor for chromoanagenesis, since there are also tumors with chromoanagenesis with intact *TP53,* and tumors with aberrant *TP53* that do not show chromoanagenesis [68,69]. In this study, we have confirmed an important role for *TP53* in the initiation of sarcomas with complex genomics, as two out of three murine MSCs carried alterations in *TP53*. This indicates, however, that also other alterations besides *TP53* can be involved. In a previous study [20], we have shown that transformed murine MSCs isolated from C57BL6/J or BALBC mice had a homozygous deletion in the locus containing *Cdkn2a* and *Cdkn2b*. These transformed murine MSCs formed osteosarcoma when injected subcutaneously into mice. In this study, we also identified a large deletion in the same gene in late passage MSCs from mouse B6_7. In osteosarcoma, alterations in *RB1* are identified in 29–47% of tumors [5,8,9,10]. RB1 activity is regulated by p16 and p15, proteins encoded by *CDKN2A* and *CDKN2B*, respectively. Moreover, mice with a conditional deletion of either *Tp53* or both *Tp53* and *Rb1* developed osteosarcomas [70]. In our COBRA-FISH karyotyping data, we also observed frequent rearrangements in chromosome 4, carrying the *Cdkn2a* and *Cdkn2b* genes. This all further supports the involvement of different pathways besides p53 towards the transformation of sarcoma with complex genomics. 

## 5. Conclusions

In summary, we have shown that transformed murine and canine MSCs provide a unique cross-species model for the identification of driver events in sarcomas with complex genomics. Spontaneously transformed murine MSCs show a plethora of genomic alterations, including copy number alterations, structural variants and point mutations, and form sarcomas in mice after subcutaneous injection. A cross-species analysis revealed that loss of p53 is a driver event in the formation of sarcomas with complex genomics. 

## Data Availability

Data is contained within the article or Supplementary Materials.

## Supplementary Materials

The following are available online at https://www.mdpi.com/2072-6694/13/5/1126/s1, Figure S1: Osteogenic and adipogenic differentiation of early passage/non-transformed murine and canine MSCs, assessed by Alizarin Red or Oil Red O staining, Figure S2: Soft agar anchorage independent growth assay of transformed murine and canine MSCs, Figure S3: Growth curve of canine MSCs isolated from 4–5 month old Labrador Retrievers, that do not transform after long-term culture, Figure S4: Coverage plots of early passage murine MSCs, Figure S5: Inter-chromosomal translocations depicted by circos style plots of (A) late passage murine MSCs and (B) late passage canine MSCs, Figure S6: Sanger sequencing chromatograms of transformed murine MSCs of exon 6 of *Trp53*, Figure S7: Sanger sequencing chromatograms of late passage canine MSCs of OSBMSC1 have a point mutation (A > T) at the site indicated by the red arrow, Figure S8: KO of exon 2–3 of *Trp53* was confirmed. (A) No p53 protein expression was detected by Western blotting in cre-mediated KO MSCs of exon 2–10 of *Trp53*, initiated by treatment with cre-recombinase (3 or 6 µM), Histon H3 was used as a loading control. Whole blots with densitometry readings can be found in Figure S8. (B) DNA from MSCs treated with cre-recombinase (6 µM) show an expected PCR-product size of 612 bp. (C) Metaphase spread of late passage Trp53 KO MSCs showing an abnormal number of chromosomes. (D) The expansion rate of MSCs from WT FVB mice treated with cre-recombinase (6 µM) is not affected compared to non-treated MSCs from WT FVB. At each data point, cells were trypsinized and counted to calculate cumulative population doubling (Cum. PD), Figure S9: Whole blots with densitometry readings from the performed Western blots, Table S1: List of copy number ratios identified in late passage murine MSCs from B6_7, Table S2: List of small nucleotide variants (SNVs) and small indels identified in late passage murine MSCs, Table S3: List of small nucleotide variants (SNVs) and small indels identified in late passage canine MSCs, Table S4: Primer sequences used in this study. All sequences are 5′ to 3′.
