# Supplementary material for: Transformed Canine and Murine Mesenchymal Stem Cells as a Model for Sarcoma with Complex Genomics
Source: Cancers (Basel). 2021 Mar 5;13(5):1126. doi: 10.3390/cancers13051126 (PMC7961539; doi:10.3390/cancers13051126)
Supplement: Supplementary file 1 [file cancers-13-01126-s001.zip › Supplemental/SF9.pdf]

A

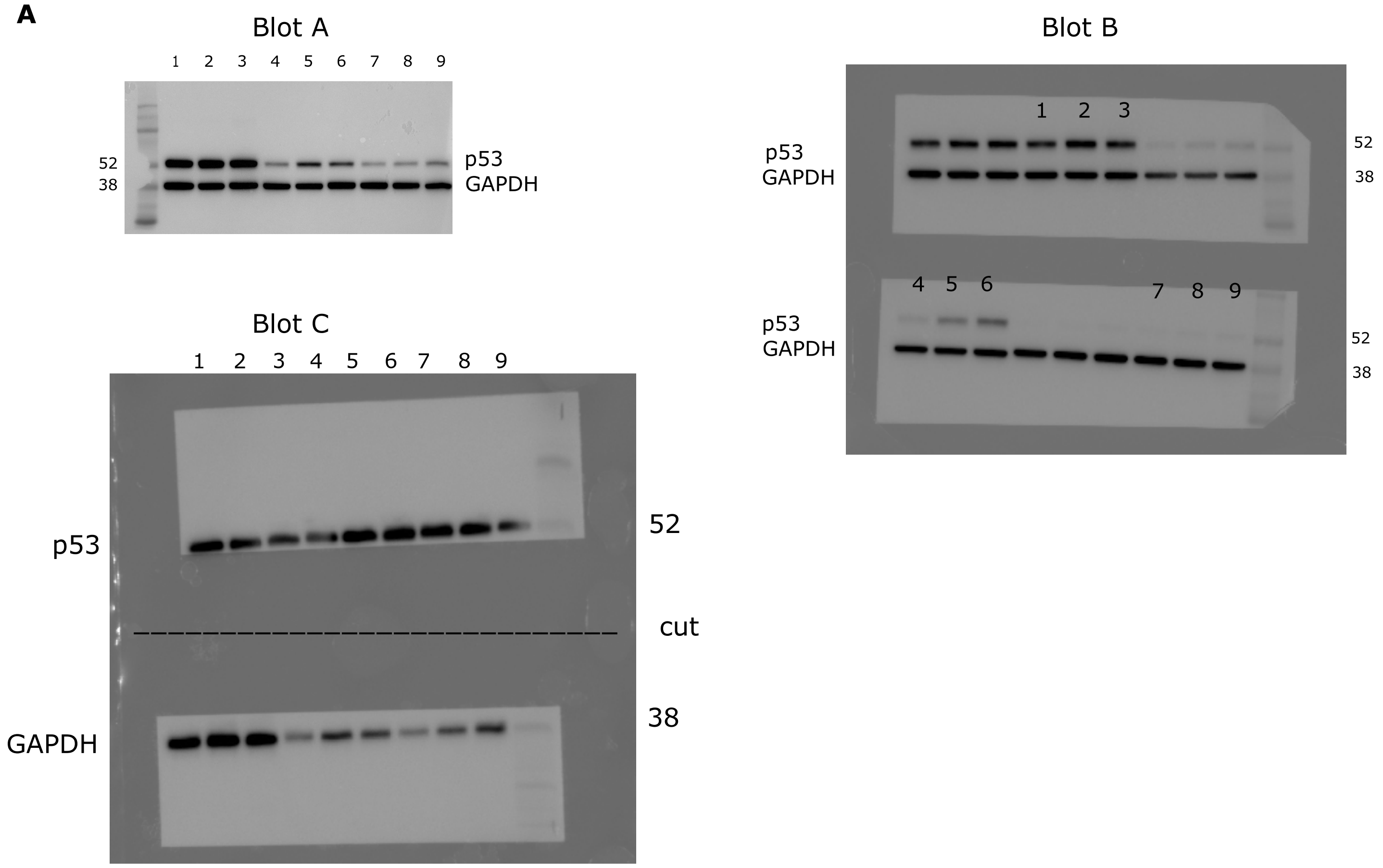

		Blot A		Blot B		Blot C	
**Nr**	**Sample**	**GAPDH**	**p53**	**GAPDH**	**p53**	**GAPDH**	**p53**
1	B6_4 (PBS)	12750650	12298440	14035600	8781840	9188398	5958020
2	B6_4 (3 µM cis)	8859107	12840084	13275040	9549203	8679460	8271690
3	B6_4 (10 µM cis)	9125079	10822107	13450720	9483360	9015134	6566186
4	B6_7 (PBS)	7842483	1408968	11773200	914560	13323467	2319096
5	B6_7 (3 µM cis)	7390915	3392187	11603680	2225840	12979266	9253517
6	B6_7 (10 µM cis)	9197928	3311462	13585920	4592560	14728957	15362706
7	B6_10 (PBS)	8568883	1223752	17410788	159975	5301401	337612
8	B6_10 (3 µM cis)	7209816	912964	16517358	269649	5113020	684526
9	B6_10 (10 µM cis)	6212365	1387779	14276493	335502	3007659	967097

B

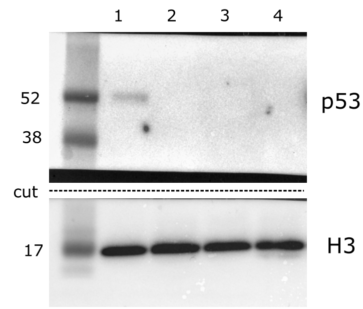

Nr.	Sample name	Intensity H3	Intensity p53
1	P53_1 (0 µM cre)	5544847	109512
2	P53_1 (3 µM cre)	5950791	936
3	P53_1 (6 µM cre)	4841991	1872
4	P53_2 (6 µM cre)	5213978	1368
