# Supplementary material for: Transformed Canine and Murine Mesenchymal Stem Cells as a Model for Sarcoma with Complex Genomics
Source: Cancers (Basel). 2021 Mar 5;13(5):1126. doi: 10.3390/cancers13051126 (PMC7961539; doi:10.3390/cancers13051126)
Supplement: Supplementary file 1 [file cancers-13-01126-s001.zip › Supplemental/ST4.pdf]

GENE	SPECIES	FORWARD PRIMER	REVERSE PRIMER
RPL8	Canine	CCATGAATCCTGTGGAGC	GTAGAGGGTTTGCCGATG
B2MG	Canine	TCCTCATCCTCCTCGCT	TTCTCTGCTGGGTGTCG
BGLAP	Canine	CTGATGGTCCTTGCCCT	CTTGGACACGAAGGTTGC
RUNX2	Canine	AACGATCTGAGATTTGTGGGC	TGTGATAGGTGGCTACTTGGG
SPARC	Canine	TCTGTATGAAAGGGATGAGGAC	GCTTCTCGTTCTCGTGGA
SPP1	Canine	GAATGCTGTGCTGACTGAGG	TGGCTATCCACATCGTCTCC
Trp53-loxsites	Murine	CACAAAAAACAGGTTAAACCCA	GAAGACAGAAAAGGGGAGGG
TP53-A668T	Canine	ATAGACTACAGGCCTGCCCA	GTCCCAGCACGTTTCCACTA
Trp53-exon6	Murine	TGGTAAGCCCTCAACACCG	ATTACAGACCTCGGGTGGCT
Trp53-exon4	Murine	ACCCTTGACTCTGGTCTCGC	ACTGACCGTCCAAGTAACAGAC
Trp53-exon5	Murine	CCCGACCCCTCTACTTCTCA	TAAGAGCCATCGGGGAGGAA
Trp53-exon7	Murine	CCTGGGCCTACCTTCTACCT	TGGGACAGGAATGGATGGGA
Trp53-exon8	Murine	ACGTAGGATGAGGGTGGCTA	GTCCCTCCTTCACCTCCTCT
