# Supplementary figures and images for: Transformed Canine and Murine Mesenchymal Stem Cells as a Model for Sarcoma with Complex Genomics
Source: Cancers (Basel). 2021 Mar 5;13(5):1126. doi: 10.3390/cancers13051126 (PMC7961539; doi:10.3390/cancers13051126)

## Osteogenic

## Adipogenic

B6\_4

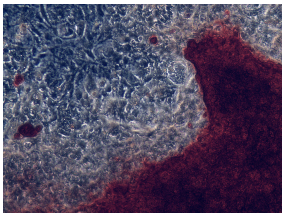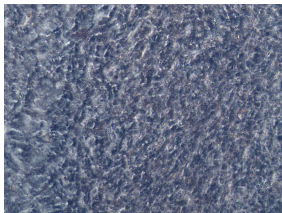

B6\_7

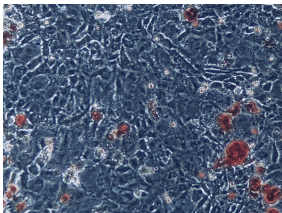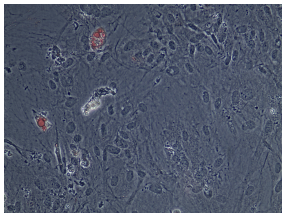

B6\_10

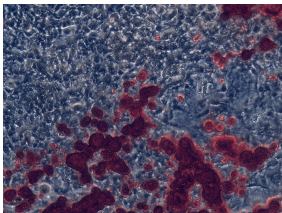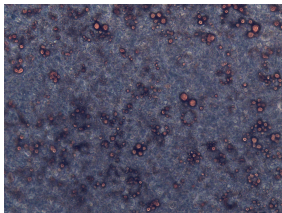

OSBMSC1

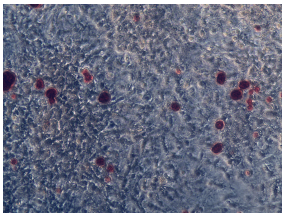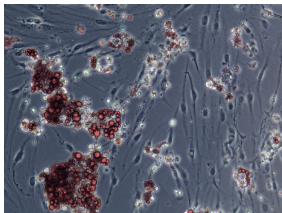

Supplement: Supplementary file 1 [file cancers-13-01126-s001.zip › Supplemental/SF1.pdf]

## Murine MSCs

---

**B6\_4**

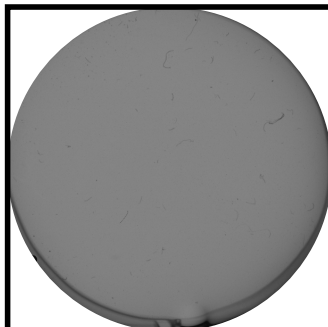

**B6\_7**

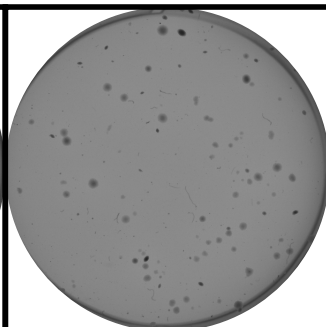

**B6\_10**

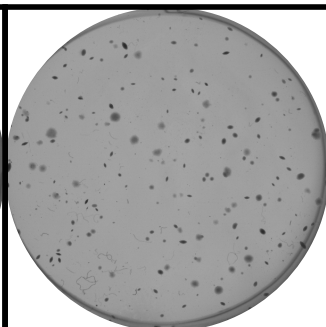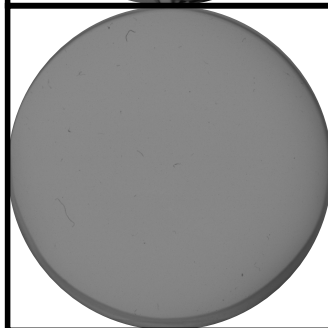

**NMRI\_2**

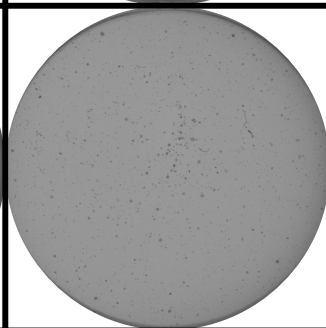

**NMRI\_3**

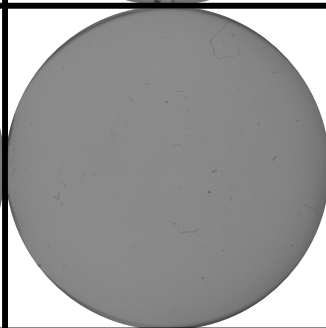

**NMRI\_9**

## Canine MSCs

---

**OSBMSC1**

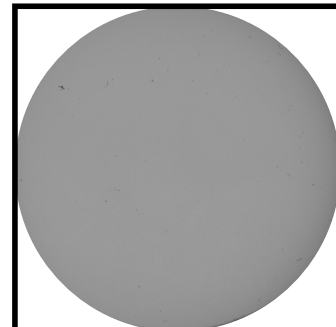

Supplement: Supplementary file 1 [file cancers-13-01126-s001.zip › Supplemental/SF2.pdf]

## Growth Curve Canine MSCs

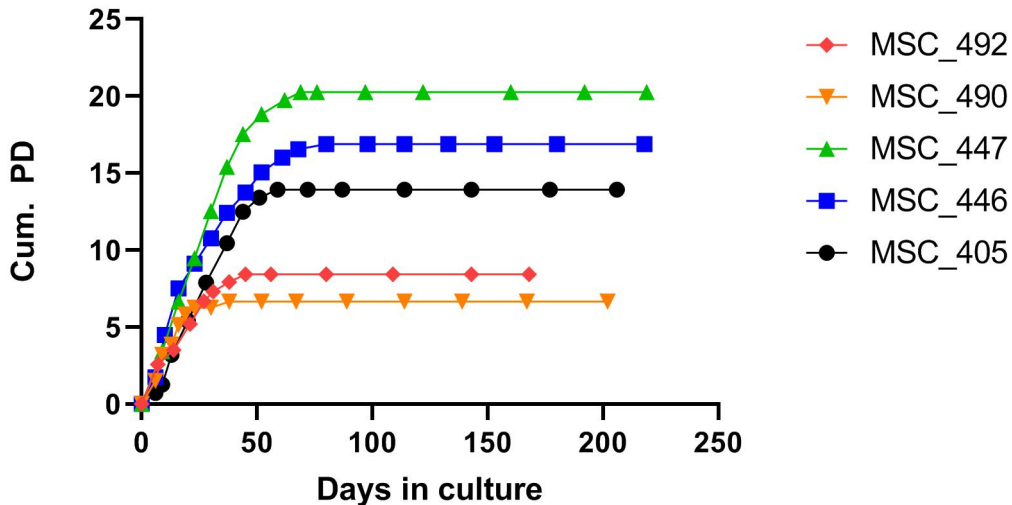

Supplement: Supplementary file 1 [file cancers-13-01126-s001.zip › Supplemental/SF3.pdf]

B6MSC4P2 Coverage

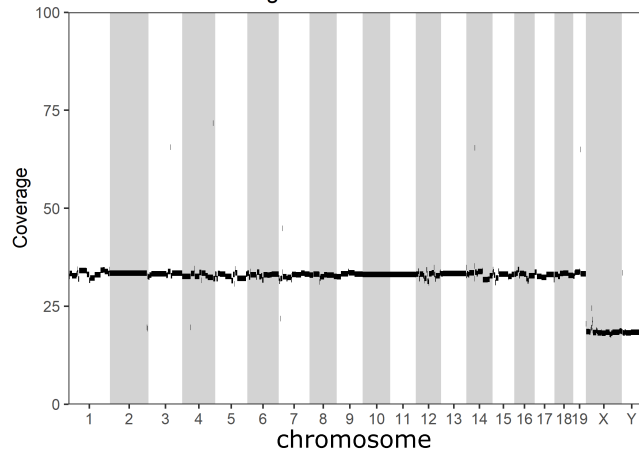

B6MSC10P3 Coverage

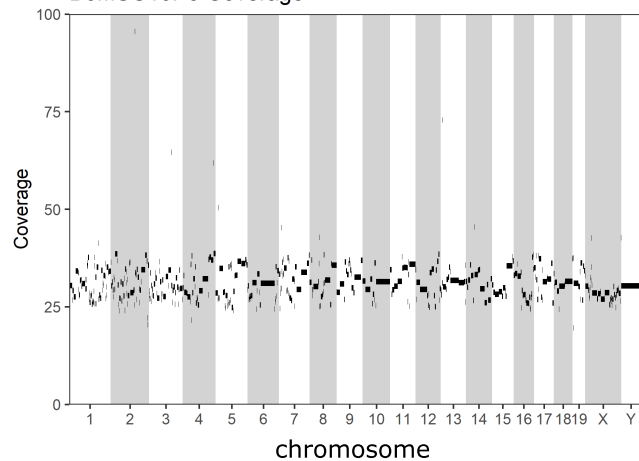

B6MSC7P2 Coverage

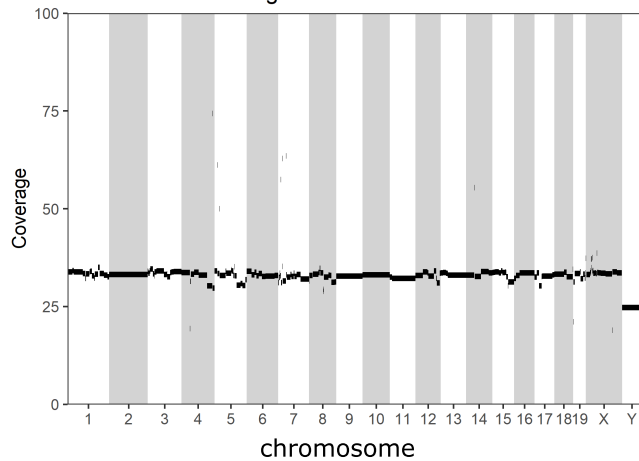

Supplement: Supplementary file 1 [file cancers-13-01126-s001.zip › Supplemental/SF4.pdf]

A B6\_4 P10

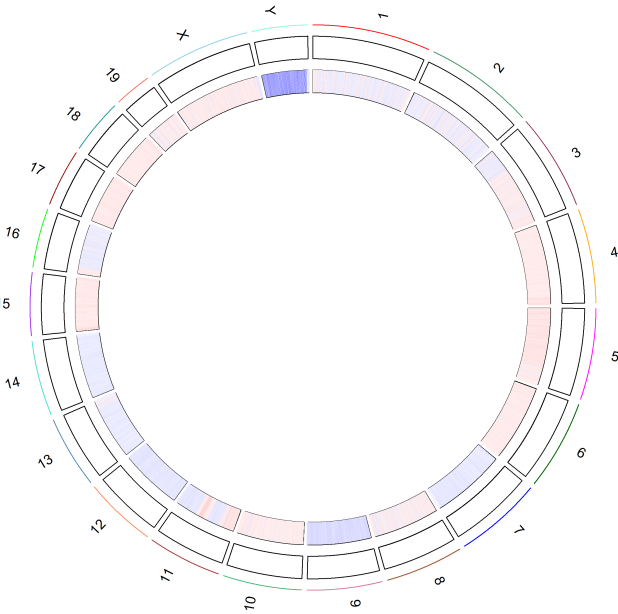

B6\_7 P15

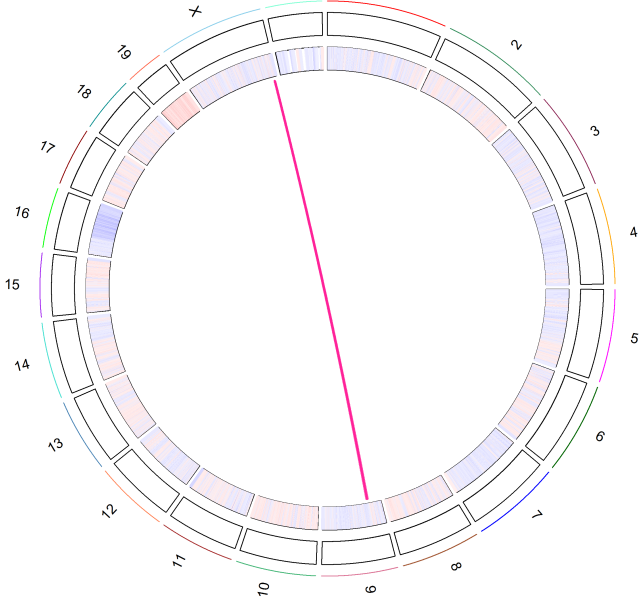

B6\_10 P13

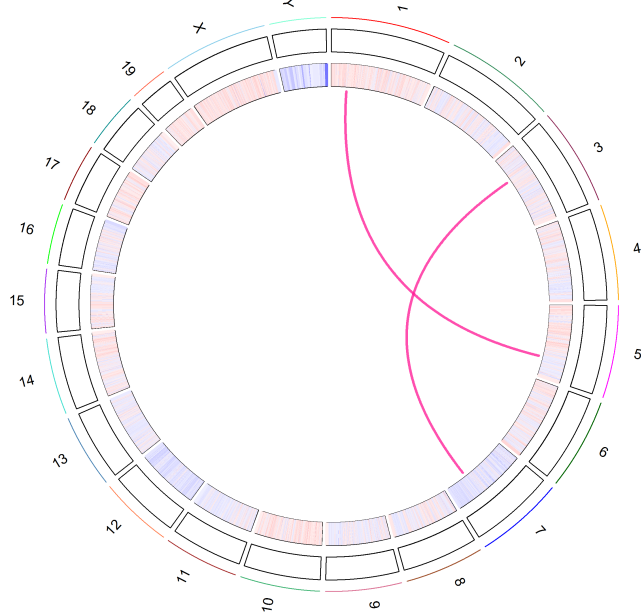

B OSBMSC1 P34

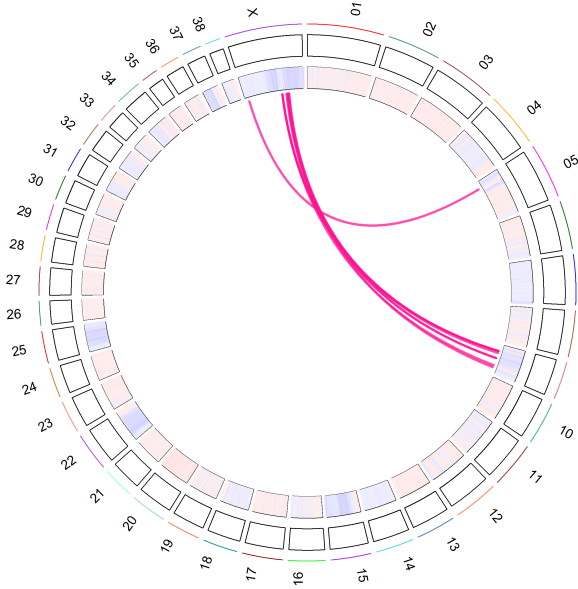

OSBMSC1 P42

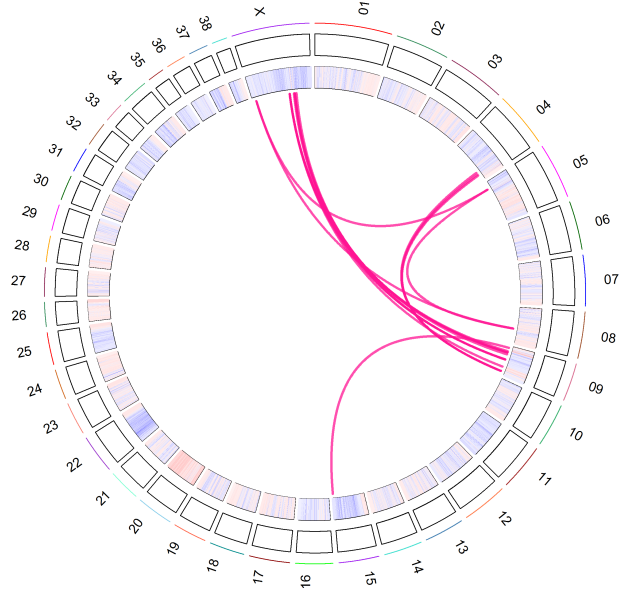

Supplement: Supplementary file 1 [file cancers-13-01126-s001.zip › Supplemental/SF5.pdf]

### B6\_4

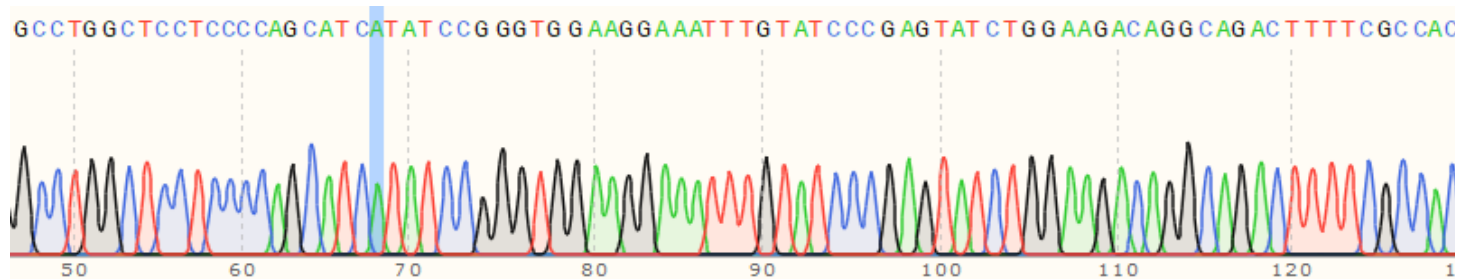

### B6\_7

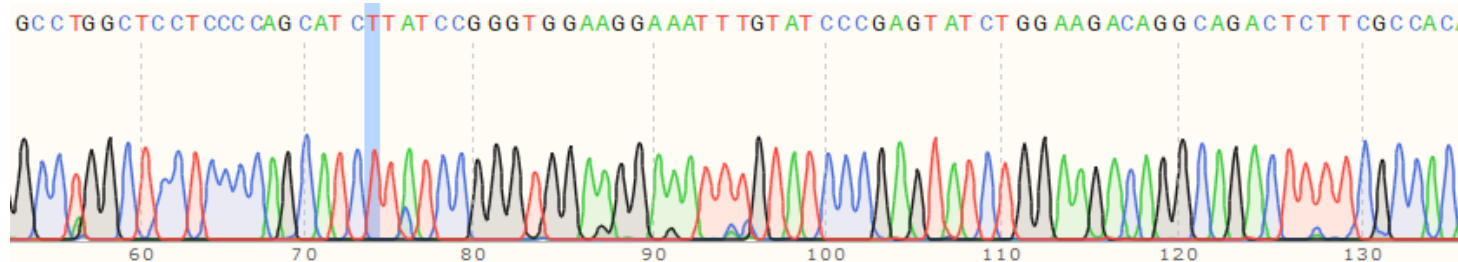

### B6\_10

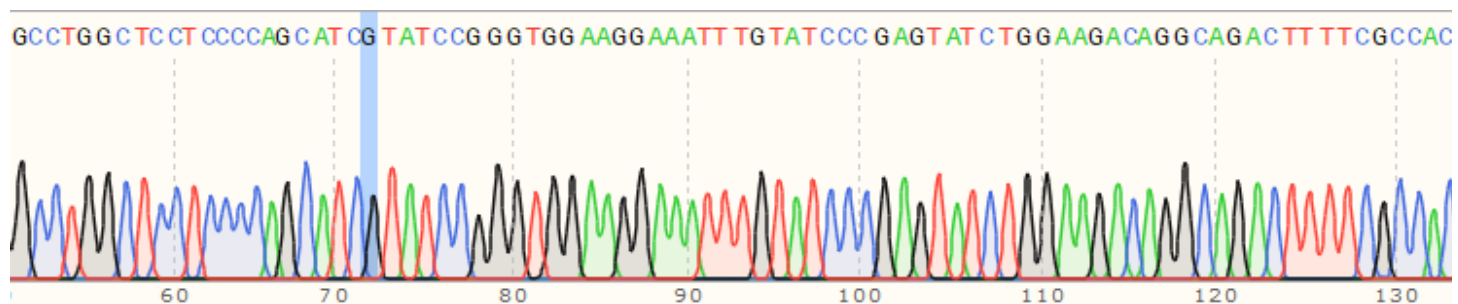

Supplement: Supplementary file 1 [file cancers-13-01126-s001.zip › Supplemental/SF6.pdf]

**OSBMSC1 tumour**

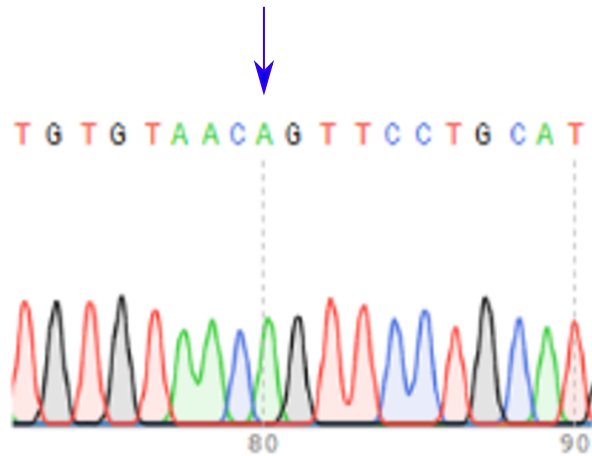

**OSBMSC1 P34**

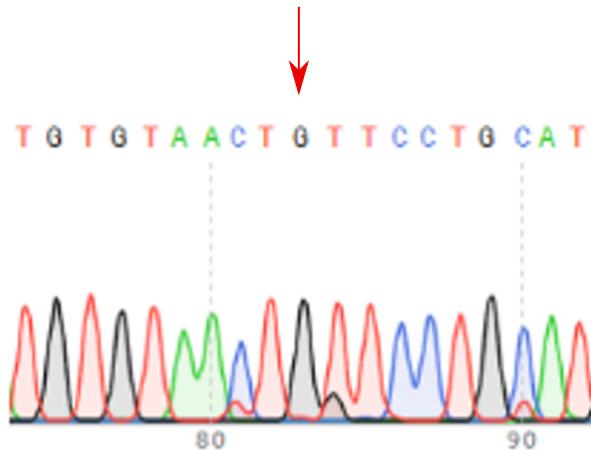

**OSBMSC1 P42**

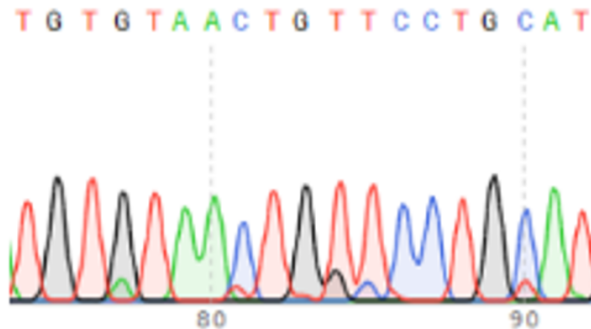

Supplement: Supplementary file 1 [file cancers-13-01126-s001.zip › Supplemental/SF7.pdf]

**A**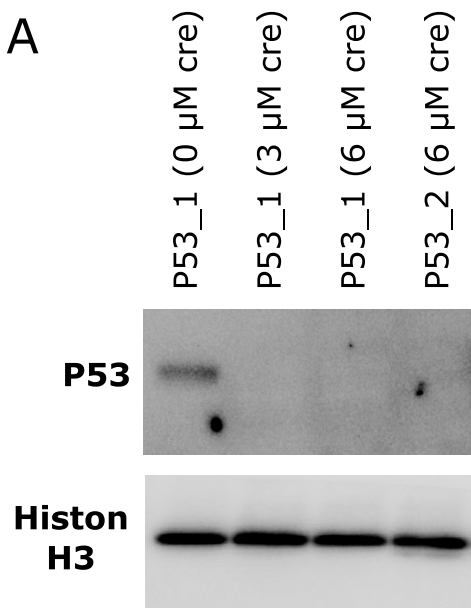**B**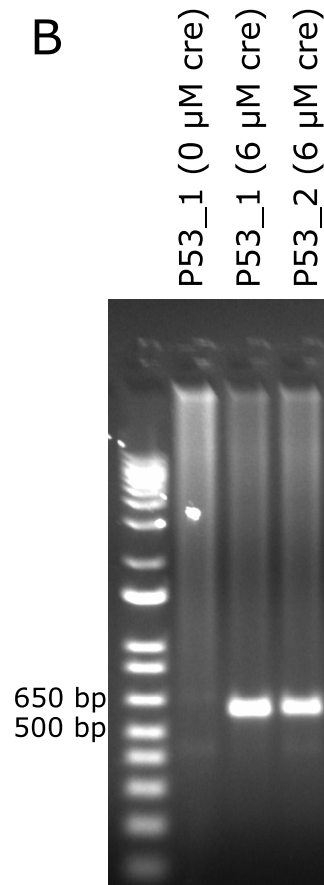**D**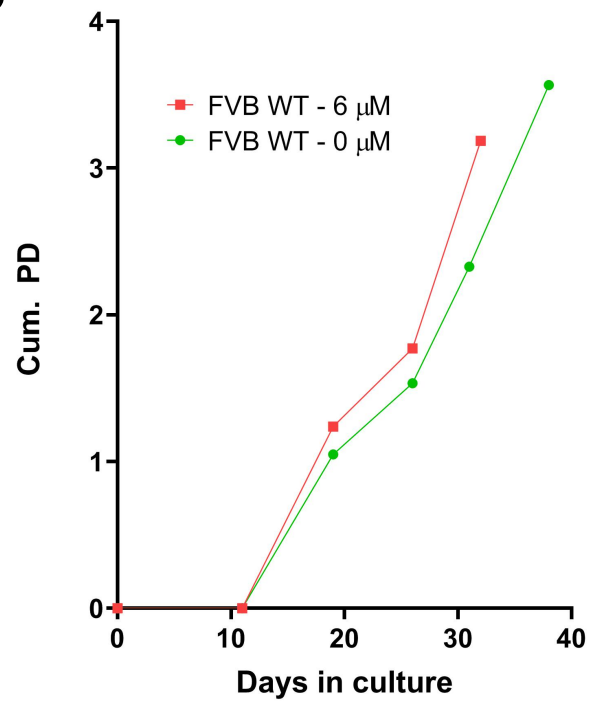**C****P53\_1 (6  $\mu$ M cre)**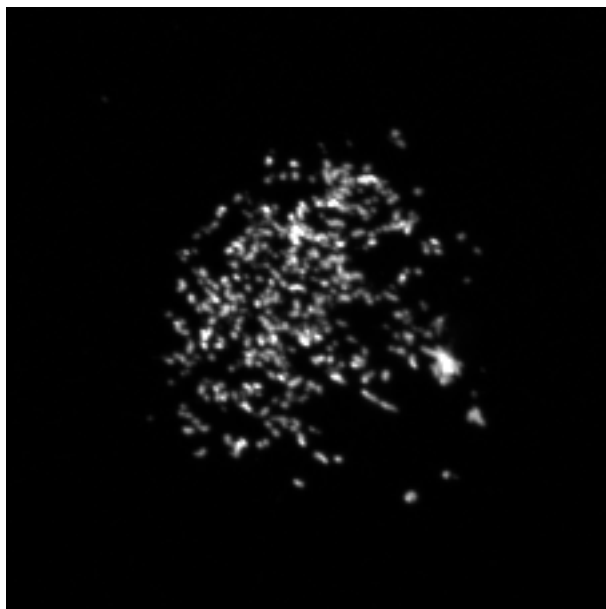**P53\_2 (6  $\mu$ M cre)**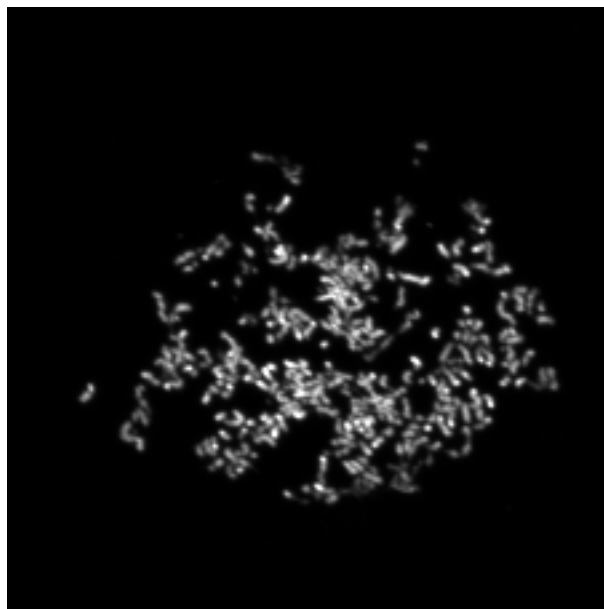

Supplement: Supplementary file 1 [file cancers-13-01126-s001.zip › Supplemental/SF8.pdf]
